# Continuous theta-burst stimulation modulates tactile synchronization

**DOI:** 10.1186/1471-2202-14-89

**Published:** 2013-08-23

**Authors:** Kevin GH Lee, Mark F Jacobs, Michael J Asmussen, Christopher M Zapallow, Mark Tommerdahl, Aimee J Nelson

**Affiliations:** 1 Department of Kinesiology, University of Waterloo, Waterloo, Ontario N2L 3G1, Canada; 2 Department of Kinesiology, McMaster University, Hamilton, Ontario L8S 4K1, Canada; 3 Department of Biomedical Engineering, University of North Carolina at Chapel Hill, Chapel Hill, North Carolina 27559, USA

**Keywords:** Temporal order judgment, Continuous theta-burst TMS, Synchronization effect, Cortical Metrics device, Primary somatosensory cortex, Tactile perception

## Abstract

**Background:**

Temporal order judgement (TOJ) is the ability to detect the order of occurrence of two sequentially delivered stimuli. Previous research has shown that TOJ in the presence of synchronized periodic conditioning stimuli impairs TOJ performance, and this phenomenon is suggested to be mediated by GABAergic interneurons that cause perceptual binding across the two skin sites. Application of continuous theta-burst repetitive TMS (cTBS) over primary somatosensory cortex (SI) alters temporal and spatial tactile perception. The purpose of this study was to examine TOJ perception in the presence and absence of synchronized periodic conditioning stimuli before and after cTBS applied over left-hemisphere SI. A TOJ task was administered on the right index and middle finger (D2 and D3) in two separate sessions in the presence and absence of conditioning stimuli (a background low amplitude sinusoidal vibration).

**Results:**

CTBS reduced the impact of the conditioning stimuli on TOJ performance for up to 18 minutes following stimulation while sham cTBS did not affect TOJ performance. In contrast, the TOJ task performed in the absence of synchronized conditioning stimulation was unaltered following cTBS.

**Conclusion:**

We conclude that cTBS suppresses inhibitory networks in SI that mediate perceptual binding during TOJ synchronization. CTBS offers one method to suppress cortical excitability in the cortex and potentially benefit clinical populations with altered inhibitory cortical circuits. Additionally, TOJ measures with conditioning stimuli may provide an avenue to assess sensory processing in neurologically impaired patient populations.

## Background

Tactile input is essential for fine motor control of the hand. Patients with impaired hand control often demonstrate abnormalities in touch processing that may contribute to their motor symptoms [1,2]. Primary somatosensory cortex (SI) is one cortical area that is clearly involved in touch perception [3-5] and importantly, has demonstrated short-term plasticity in a number of repetitive transcranial magnetic stimulation (rTMS) studies [6-10].

Previous studies suggest that SI is involved in temporal processing of tactile information. In focal hand dystonia, functional [1] and anatomical abnormalities in SI [11,12] are present. These individuals also demonstrate impaired temporal discrimination threshold (TDT), which is defined as the ability to detect the presence of one versus two stimuli when the pair is delivered over the skin and separated by a varied time interval [9,13-15]. TDT impairments are greatest when lesions affect SI compared to the frontal, temporal and occipital cortex [15]. However, other cortical areas are considered important in TDT processing, including the prefrontal cortex, inferior parietal lobe, the basal ganglia, cerebellum, the pre-supplementary motor area and anterior cingulate [14]. Temporal order judgment (TOJ) represents another feature of tactile temporal processing; in this task, subjects are required to detect the temporal order of two sequential stimuli delivered across skin sites. In humans, it remains unclear which cortical areas are involved in processing TOJ. There is some evidence in animal studies, however, suggesting the role of SI in TOJ. One study reported an increase in c-Fos expression, a task-relevant neural activation marker in SI of mice, following a temporal order judgment task performed with tactile stimuli delivered to the whiskers [16]. Specifically, c-Fos was increased in the barrel fields of SI following a TOJ task in which mice were trained to detect the order of two tactile air-puff stimuli by orienting their head towards the first or second stimulus [16]. These results suggest that SI may play a role in TOJ processing.

A perceptual phenomenon called the ‘synchronization effect’ (TOJ-S) occurs when TOJ is performed in the presence of low amplitude background synchronized vibration (low frequency flutter or 25 Hz) delivered to both skin sites such that TOJ thresholds are impaired in healthy individuals by a factor of 2 to 4 times [17,18]. The impact of TOJ-S is thought to occur by the co-activation of adjacent and/or near-adjacent cortical ensembles in SI that results from conditioning tactile stimuli applied synchronously to adjacent digits. The co-activation of these cortical ensembles perceptually bind adjacent skin sites such that a stimulus presented at one site evokes a response in the adjacent cortical representation, and this leads to impaired TOJ performance [18]. Inhibitory interneurons are thought to participate in TOJ-S as it is well documented that inhibition plays a role in cortical synchronization [19,20]. For example, there is growing evidence that deficiencies in GABA play a role in autism [21] and the TOJ synchronization effect is abolished in these individuals [22]. Dopaminergic neurotransmitter systems may also contribute such that Parkinson’s patients on L-dopa do not demonstrate the synchronization effect but show typical impairments when off medication [17]. In the present study, we investigate the role of SI in TOJ processing in the presence and absence of the synchronization effect.

One method to investigate the role of SI in TOJ processing is via the application of continuous theta-burst stimulation (cTBS) [23]. Previous studies observed impairments in TDT for 5 to 18 minutes following cTBS over SI [6,9]. Such impairments in TDT are not observed when cTBS was applied to the dorsal lateral prefrontal cortex or lateral cerebellum [6]. Similarly, tactile two-point discrimination is also impaired for up to 18 minutes following stimulation over SI [9]. Previous reports examining SI physiology demonstrate that cTBS over SI suppresses ipsilateral somatosensory evoked potentials (P25/N33) for 13 minutes following stimulation [24]. Further, decreased oxy-hemoglobin concentrations in contralateral SI and primary motor cortex (M1) are also observed following cTBS over SI [25]. In the present study, we investigate the influence of cTBS over SI on TOJ and TOJ-S. Psychophysical measures were obtained from the right hand before and for up to 34 minutes following real and sham cTBS over left-hemisphere SI [9].

## Methods

### Participants

Sixteen healthy adults were recruited (mean age = 23.1 ± 5.2 years, range 19 – 36 years, 5 males). For experiment 1, eight subjects (mean age = 26.5 ± 5.4 years, range 19 – 36 years, 3 males) participated in two sessions separated by a minimum of one week. For experiment 2, eight subjects (mean age = 19.7 ± 1.4 years, range 19 – 23 years, 2 males) participated in a single session. There was no participant overlap between experiments. All participants were right handed determined using a subsection of the Edinburgh Handedness Inventory [26]. Subjects wore earplugs and headphones to minimize auditory cues during the experiments. All participants provided written consent and the study was approved by the Office of Research Ethics at the University of Waterloo and conformed to the Declaration of Helsinki.

### Electromyography (EMG) recording

Measurements of muscle activity were recorded using 9 mm diameter Ag-AgCl surface electrodes. The active electrode was placed over the muscle belly of the right first dorsal interosseous muscle (FDI) and the reference electrode was placed over the metacarpophalangeal joint of the right index finger. EMG was amplified at 1000× gain, bandpass filtered (2 Hz – 2.5 kHz, Intronix Technologies Corporation Model 2024F, Bolton, Ontario, Canada), and digitized (5 kHz, Micro 1401, Cambridge Electronics Design, Cambridge, UK). Signal software (v4.02, Cambridge Electronic Design Limited, Cambridge, UK) was used to acquire and analyze EMG data. Data was stored on a computer for analysis purposes.

### TMS and neuronavigation

TMS was delivered with a biphasic waveform through a MagPro stimulator (MCF-B65; Medtronic, Minneapolis, MN, USA) connected to a 90 mm outer diameter figure-of-eight coil. For all TMS, the handle was oriented backwards and laterally at a 45 degree angle to the mid-sagittal line such that the current induced in the cortex flowed in an anterior to posterior followed by posterior to anterior (AP-PA) direction. The motor hotspot was defined as the location in the left hemisphere that elicited a MEP in the relaxed right FDI muscle. Active motor threshold (AMT) was determined at this location and defined as the lowest intensity required to evoke MEPs ≥ 400 μV in 5 out of 10 consecutive trials during 10% maximum voluntary contraction (MVC) of the right FDI muscle. MVC was determined by having participants abduct their right index finger against an immovable post with maximal force. Participants maintained 10% MVC using EMG feedback from the FDI muscle displayed visually on an oscilloscope. Brainsight Neuronavigation software (Rogue Research, Montreal) was used to mark the location of the M1 motor hotspot. SI was defined as a point 2 cm posterior to the M1 motor hotspot [9,24,27] as measured using Brainsight Neuronavigation. CTBS was applied over SI using the 600 biphasic pulse protocol [23,28] at 80% AMT in the AP-PA current direction [9,27]. The orientation and position of the coil were marked using the Brainsight software to ensure theta-burst stimulation was delivered with minimal spatial variability.

### Experimental paradigm

Subjects were seated comfortably in a chair with their left hand resting on a computer touch pad and their right hand placed on the Cortical Metric Device, version CM-4 [29]. Both the computer laptop and the CM-4 device were positioned at a comfortable arm level in front of the participants. The CM-4 is equipped with 4 circular probes that are located on the surface of each individual rotatory cylindrical disk [29]. Each disk was rotated independently to adjust for different finger lengths for each participant. Digits 2 through 5 of the right hand were comfortably rested on the surface of the circular probes such that a single probe (5 mm diameter) maintained contact with the glabrous pad of each digit. The finger tips were locked in place prior to each TOJ task. The probes were further indented 500 μm prior to stimulation onset to ensure adequate skin contact across the surface area of the probe. An optical position sensor was attached to each circular probe to provide feedback to the CM-4 device to ensure that the contact force of each fingertip was constant throughout the TOJ task [29].

### Experiment 1: CTBS influence on TOJ and TOJ-S

#### Temporal order Judgement (TOJ)

**Figure 1:**
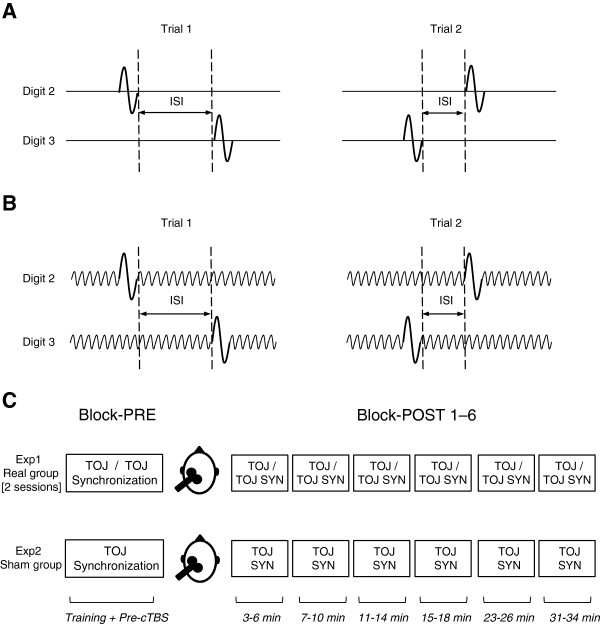
**Experimental tasks and timeline. (A)** Temporal order judgement (TOJ). Two sequential vibro-tactile stimuli were delivered in random order to digit two and digit three. Two trials shown with subject response from the first trial resulting in a decrease in the interstimulus interval (ISI). **(B)** Temporal order judgement with synchronization (TOJ-S). 25 Hz conditioning stimulus delivered concurrently with TOJ task. Two trials shown with subject response from the first trial resulting in a decrease in the interstimulus interval (ISI). **(C)** Timeline for Experiment 1 and 2.

TOJ was performed on digits 2 and 3 of the right hand. A single TOJ trial delivered a vibro-tactile stimulus (25 Hz, 40 ms and 200 μm) to the volar surface of the second and third digit tips on the right hand separated by an interstimulus interval (ISI) (see Figure 1A). The participant was queried to identify which stimulus occurred first (i.e. the 2^nd^ or 3^rd^ digit) and respond as quickly as possible by making a key press with the left hand; left key = 2^nd^ digit, right key = 3^rd^ digit. The digit selected to receive the first stimulus was randomized on a trial-by-trial basis. The ISI was initially set at 150 ms [17,18] and was subsequently altered by a step size of 15% based on the accuracy of the participant’s response. TOJ was performed in blocks of 20 trials. During the first 10 trials, a 1 up/ 1 down tracking paradigm was used, allowing a single correct answer to cause a 15% reduction of the ISI in the subsequent trial. If an incorrect response was made, the ISI increased by 15% in the following trial. For the last 10 trials, a 2 up/ 1 down tracking algorithm was employed in which two correct responses were required to decrease the ISI by 15%. The combination of these two tracking algorithms enables rapid and reliable determination of each subject’s TOJ thresholds [17,18]. The inter-trial interval was set at 5 seconds. The threshold for TOJ was defined as the average ISI measured from the last five trials within each block (trials 16 to 20) as performed elsewhere [17,18,22].

#### TOJ Synchronization (TOJ-S)

TOJ-S was performed on digits 2 and 3 of the right hand. Specifically, a conditioning sinusoidal vibration (25 Hz, 20 μm) was applied to digits 2 and 3 before, concurrently and after the TOJ stimulus pair [18]. The task requirements were identical to the TOJ task in that participants were queried to report which stimulus occurred first within the pair. Twenty TOJ-S trials were performed using the identical 1 up/ 1 down and 2 up/ 1 down structure used for the TOJ task. TOJ-S thresholds were taken as the average of the last five trials within a block (trials 16–20). A schematic of a TOJ-S task is shown in Figure 1B.

#### Experiment Timeline

TOJ and TOJ-S were measured in different sessions separated by a minimum of one week. Five participants performed TOJ first while the other three participants performed TOJ-S first. Within each session, the psychophysical task was performed in 7 blocks (20 trials each) before (*T*_*0*_) and after cTBS at 3–6 min (*T*_*1*_), 7–10 min (*T*_*2*_), 11*–*14 min (*T*_*3*_), 15–18 min (*T*_*4*_), 23–26 min (*T*_*5*_), and 31–34 min (*T*_*6*_), in line with our previous report [9]. The timeline is depicted in Figure 1C. Prior to performing *T*_*0*_ participants completed training trials that required five consecutive trials to be performed correctly. During training, visual feedback was displayed on the computer; “Good job” was presented following a correct response and “Please try again” was presented if an incorrect response was made. Once performance criteria on the training trials were met, the pre-cTBS block began. No feedback was given during the 7 testing blocks.

### Experiment 2: Sham cTBS on TOJ-S

Participants performed the TOJ-S task as described above. The protocol was identical to the TOJ-S protocol performed by the real group. The timeline is shown on Figure 1C. Prior to performing *T*_*0*_ participants also completed training trials that required five consecutive trials to be performed correctly. Once performance criteria on the training trials were met, *T*_*0*_ began. No feedback was given during the 7 testing blocks. The sham stimulation delivered the real cTBS protocol. However, the cTBS coil was placed over SI and rotated 90 degrees such that the handle of the coil pointed vertically upward away from the scalp. The coil maintained scalp contact during stimulation.

### Data analysis

To assess the effects of cTBS on TOJ versus TOJ-S over time, post-cTBS values (*T*_*1*_*, T*_*2*_*, T*_*3*_*, T*_*4*_*, T*_*5*_*, T*_*6*_) were normalized to pre-cTBS values (*T*_*0*_) for each task, respectively. A two-way repeated measures analysis of variance (ANOVA) with within-subject factors ‘TIME’ (6 levels: 3–6 min (*T*_*1*_), 7–10 min (*T*_*2*_), 11*–*14 min (*T*_*3*_), 15–18 min (*T*_*4*_), 23–26 min (*T*_*5*_), and 31–34 min (*T*_*6*_)) and ‘TASK’ (2 levels: TOJ, TOJ SYN) was performed. Two separate one-way repeated measures ANOVA with within-subject factor ‘TIME’ (7 levels: 0 min (*T*_*0*_), 3–6 min (*T*_*1*_), 7–10 min (*T*_*2*_), 11*–*14 min (*T*_*3*_), 15–18 min (*T*_*4*_), 23–26 min (*T*_*5*_), and 31–34 min (*T*_*6*_)) were performed for TOJ and TOJ-S, respectively. A priori hypotheses were tested using contrast estimations and Bonferroni corrected for cTBS effects on TOJ (4 comparisons: *T*_*0*_ vs *T*_*1*_, *T*_*0*_ vs *T*_*2*_, *T*_*0*_ vs *T*_*3*_, *T*_*0*_ vs *T*_*4*_). No hypothesis was created for TOJ-S. Post-hoc analysis was performed using Dunnett’s t-test to test for differences following cTBS. To assess the effects of cTBS on TOJ-S (sham group) over time, a one-way repeated measures ANOVA with within-subject factor ‘TIME’ (7 levels: *T*_*0*_, 3–6 min (*T*_*1*_), 7–10 min (*T*_*2*_), 11*–*14 min (*T*_*3*_), 15–18 min (*T*_*4*_), 23–26 min (*T*_*5*_), and 31–34 min (*T*_*6*_)) was performed. All statistical analysis was performed using SAS 9.2 Windows software (SAS Institute Inc., Cary, North Carolina, US). Significance level was set at *p* ≤ 0.05.

## Results

### Experiment 1: cTBS influence on TOJ and TOJ-S

All participants successfully completed the experiment. The group-averaged AMT (with standard deviation) for TOJ was 45.4 ± 7.6% of the maximum stimulator output (MSO) with cTBS delivered at 36.3 ± 6.1% MSO. The mean AMT for TOJ-S was 43.4 ± 8.2% MSO of the stimulator output with cTBS delivered at 34.8 ± 6.6% MSO. A paired *t*-test (one-tail) revealed no significant differences between the MSO for TOJ and TOJ-S (*p* = 0.09).

**Figure 2:**
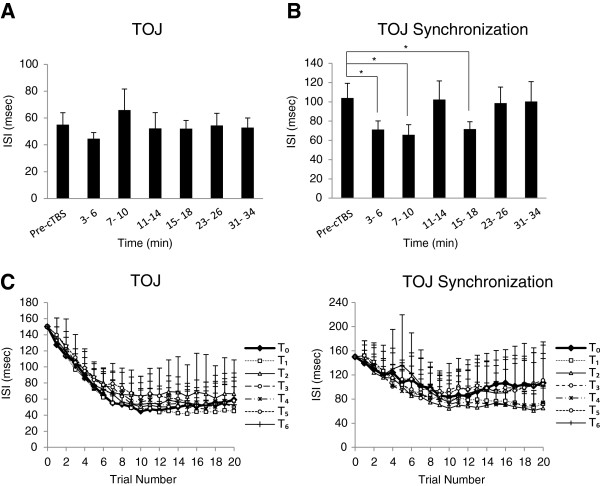
**Experiment 1: cTBS influence on TOJ and TOJ-S. (A)** Group-averaged TOJ (with standard errors) before and at each time block following cTBS. * p ≤ 0.05. Time blocks measured *T*_*0*_, 3–6 min (*T*_*1*_), 7–10 min (*T*_*2*_), 11*–*14 min (*T*_*3*_), 15–18 min (*T*_*4*_), 23–26 min (*T*_*5*_), and 31–34 min (*T*_*6*_). **(B)** Group-averaged TOJ-S (with standard errors) before and at each time block following cTBS. * p ≤ 0.05. **(C)** Left: Group-averaged performance for each trial in each time block for the TOJ condition. Right: Group-averaged performance for each trial in each time block for the TOJ-S condition.

Two-way ANOVA revealed a significant main effect of TASK (*F*_(1, 7)_ = 8.12, *p* = 0.02), no effect of TIME (F _(5, 35)_ = 1.16, p = 0.35) and no interaction between TASK and TIME (*F*_(5, 35)_ = 1.55, *p* = 0.19). Two separate one-way repeated ANOVAs were performed for each task (TOJ, TOJ-S) with factor ‘TIME’ (*T*_*0*_, *T*_*1*_, *T*_*2*_*, T*_*3*_*, T*_*4*_*, T*_*5*_*, T*_*6*_). For TOJ, ANOVA revealed no significant main effect of TIME (*F*_(6, 42)_ = 0.78, *p* = 0.59). A paired *t*-test with Bonferroni corrected contrasts (corrected for four comparisons) was performed to compare pre-cTBS values (*T*_*0*_) to post-cTBS values (*T*_*1*_, *T*_*2*_*, T*_*3*_*, T*_*4*_) individually for up to 18 minutes following cTBS. Performance was not significantly different between all four blocks versus *T*_*0*_. The group-averaged data (with standard errors) for TOJ are shown in Figure 2A. For TOJ-S, ANOVA revealed a significant main effect of TIME (*F*_(6, 42)_ = 2.27, *p* = 0.05). Post-hoc analysis using Dunnett’s t-test revealed that TOJ-S values were significantly lower at time blocks *T*_*1*_ (3–6 min, p = 0.049), *T*_*2*_ (7–10 min, p = 0.022) and *T*_*4*_ (15–18 min, *p* = 0.05). The group-averaged data (with standard errors) for TOJ-S are shown in Figure 2B, respectively. Group-averaged trial-by-trial TOJ and TOJ-S performance is shown in Figure 2C. Note that the improvements for TOJ and TOJ-S performance begins to plateau at ~ trial 10 as shown in previous experiments [9,18] and that the effects of cTBS on TOJ-S occur during optimal performance. To investigate whether cTBS significantly altered performance during non-optimal performance (trials 6 through 10) and as threshold values were approached (trials 11 through 15) two one-way ANOVAs with factor TIME were performed. These analyses revealed no significant main effect of TIME for non-optimal performance (trials 6 to 10, *F*_(6, 42)_ = 0.57, *p* = 0.75) and no significant main effect of TIME as threshold values were approached (trials 11 to 15, *F*_(6, 42)_ = 0.96, *p* = 0.46).

### Experiment 2: Sham cTBS on TOJ-S

**Figure 3:**
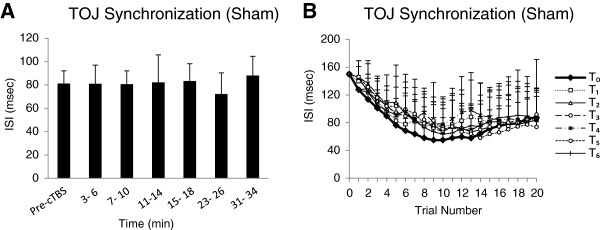
**Experiment 2: Sham cTBS on TOJ-S. (A)** Group-averaged TOJ-S (with standard errors) before and at each time block following sham cTBS. * p ≤ 0.05. Time blocks measured *T*_*0*_, 3–6 min (*T*_*1*_), 7–10 min (*T*_*2*_), 11*–*14 min (*T*_*3*_), 15–18 min (*T*_*4*_), 23–26 min (*T*_*5*_), and 31–34 min (*T*_*6*_). **(B)** Group-averaged performance for each trial in each time block following sham cTBS for the TOJ-S condition.

All participants completed the experiment successfully. The mean AMT for the TOJ-S sham group was 53 ± 7.5% MSO with sham cTBS delivered at 42 ± 5.8% MSO. The ANOVA revealed no significant effect of TIME (*F*_(6, 42)_ = 0.35, *p* = 0.90). Figure 3A displays the group-averaged TOJ-S (with standard errors) before and following sham cTBS. Group-averaged trial-by-trial TOJ-S performance before and following sham cTBS is shown on the right graph of Figure 3. Note that sham TOJ-S performance improvement plateaus at ~ trial 10.

## Discussion

The present study investigated the influence of cTBS over left-hemisphere SI on TOJ performance and the TOJ synchronization effect in the contralateral hand. Novel findings indicate that cTBS reduced the TOJ synchronization effect for up to 18 minutes while sham cTBS had no such effect. We attribute cTBS effects to changes in the excitability of neural activity within SI. We discuss these findings and their neural mechanisms below.

In the present study, TOJ performance was unaltered following cTBS which questions the role of SI in TOJ processing. This finding was unexpected as previous studies showed changes in tactile perception after suppression-inducing protocols such as low frequency repetitive TMS [30,31] and cTBS [6,9]. However, it should be noted that TDT and TOJ tasks are not identical. Therefore, cTBS may act differently on the populations of neurons that mediate each of these percepts [6,9]. Alternatively, the lack of change in TOJ may relate to cTBS technical parameters such as intensity and the direction of induced current flow, which are known to determine cTBS effects [32-34]. For instance, cTBS delivered over the primary motor cortex (M1) at 80% AMT yields different results in MEP amplitudes when delivered at 70% RMT [35]. Another explanation may be that other cortical areas may be dominant in the TOJ task, including the secondary somatosensory cortex [36,37], parietal cortex [38,39], anterior cingulate, supplementary motor areas [14,15] and the cerebellum [40], which may compensate for changes in SI excitability induced by cTBS. There is also growing evidence for the specialized role of the superior temporal gyrus in tactile temporal perception [41]. Most recently, functional magnetic resonance imaging data indicate that prefrontal and parietal cortices may play an integral part in TOJ [42]. Hence, contributions from different cortical or subcortical areas may suggest the complexity of tactile TOJ.

Following cTBS, we observed a reduction of the TOJ-S effect. TOJ-S thresholds were reduced for up to 18 minutes. Significant reduction of the TOJ-S effect occurred from 3 to 10 minutes and again from 15–18 minutes following cTBS. The maximum effect was observed from 7–10 min following cTBS, which is the timeframe for maximal physiological effects of cTBS seen elsewhere [24,28,43]. We observed that the TOJ-S effect is abolished from 7 to 10 minutes following cTBS such that thresholds were not different from TOJ pre-cTBS values (paired t-test, TOJ baseline versus TOJ-S at *T*_*2*_, p = 0.21). The time varying effect of cTBS on TOJ is also similar to the effects on TDT [9]. Specifically, both studies observed significant impairments immediately following cTBS, followed by no significant change from 11 to 14 minutes and followed again by significant perceptual impairments from 15 to 18 minutes [9]. Further, both studies indicate that cTBS effects persist for up to 18 minutes and not at later time blocks. Exposing such variability in the time course of cTBS effects may be a result of the frequent sampling intervals used in our study (i.e. every 3 minutes without inter-block breaks).

The mechanisms that underpin TOJ and TOJ-S are not fully understood although GABAergic activity via lateral inhibition across cortical columns and in-field inhibition within cortical columns likely mediates these percepts. For the TOJ task, the somatosensory cortex provides information about the loci of the two tactile stimuli, and in the absence of the synchronized conditioning stimulus, this information is robustly delivered. In the presence of periodic and synchronous conditioning stimuli to D2 and D3, it has been proposed that the evoked response of the cortical representations of D2 and D3 become functionally linked in a manner that a tap to one digit results in a response at both sites and a consequent degradation of spatial resolution between digit representations [18,22]. Recent observations from in vivo non-human primate studies support that idea [44], and although the mechanisms of this synchronization effect are not fully understood, GABAergic mediated activity (e.g., lateral inhibition) is a necessary component. Stimulation of afferent fibers creates excitation in corresponding cortical columns that evoke lateral inhibition between the excited columns. The amount of lateral inhibition depends on the magnitude and duration of the initial excitation within the cortical columns [45,46]. Lateral inhibition dissipates over time, resulting in decreased lateral inhibition received from neighbouring columns [47]. We speculate that correct TOJ performance occurs when lateral inhibition dissipates to allow the cortical columns receiving the second stimulus in the TOJ pair to be excited. There is some evidence that lateral inhibition is also fundamental for the TOJ-S effect. Patients with autism demonstrate a narrowing of neuropil space between minicolumns, an effect associated with a reduction in GABAergic interneurons [48] that mediate lateral inhibition. In contrast to control subjects, autistic patients do not demonstrate the TOJ-S effect [22]. Further, the absence of the TOJ-S effect in migraineurs and concussed individuals has been postulated to be the result of an imbalance between cortical excitation and GABA mediated inhibition [49,50]. In addition to lateral inhibitory mechanisms that function across the columns, in-field inhibition occurs within cortical columns whereby the period of initial excitation is followed by a period of inhibition that persists from ~ 60 to 100 ms [47]. We speculate that this type of inhibition may be particularly relevant to the TOJ-S task whereby the low-amplitude background vibration creates synchronous excitation in adjacent cortical columns. For TOJ to be performed in the presence of such synchronous vibration, the excitation of the cortical columns evoked by the second stimulus in the TOJ pair must exceed both in-field inhibition created by the low-amplitude vibration and the lateral inhibition.

Although the mechanisms by which cTBS alters neural activity are not fully understood, there is evidence to indicate that inhibitory networks within SI are suppressed. Previous work demonstrates that late sub-components of high frequency oscillations (HFO) evoked potentials from SI, which are associated with GABA inhibitory interneurons in superficial layers within SI [51,52], are suppressed by cTBS over SI at 15 min [28]. In the present study, cTBS is likely to have suppressed lateral and/or in-field inhibitory circuits that mediate tactile perceptual binding across cortical columns, thereby reducing the synchronization effect for up to 18 minutes following stimulation.

The present research demonstrates that cTBS alters TOJ synchronization performance and we believe that these changes are not attributed to cTBS altering learning processes. CTBS affects motor learning in healthy individuals [53,54] and in post-stroke patients [55]. Further, cTBS has shown to degrade timing accuracy of a sensorimotor synchronization task [56]. However, in rats, cTBS does not alter the learning of a tactile discrimination task [57]. We implemented approaches to minimize such learning in the present study. First, training trials were presented in advance of the testing trials. Such training trials required subjects to correctly complete 3 blocks of 5 consecutive correct trials prior to data acquisition. Second, thresholds were calculated as the average of the last five trials within each block, that is, from trials 16 through 20. Performance during TOJ plateaus at ~ trials 10 and beyond [18,22]. Therefore, we are using data only from trials in which there is no further change in performance.

In summary, we found that continuous theta-burst stimulation over the primary somatosensory cortex reduced the synchronization effect that led to an improvement in TOJ performance. There were no significant changes to TOJ performance when cTBS was delivered over SI. This study adds direct evidence that cTBS induces temporal changes in the SI that lead to altered tactile perception [6,9]. It has provided a more refined hypothesis regarding the underlying mechanisms of tactile perception that can be tested in future studies.

## Competing interests

KL, MJ, MA, CZ and AJN have no competing interests. Mark Tommerdahl is co-inventor of the tactile stimulator used in the study and co-founder of Cortical Metrics, LLC, which has a licensing agreement with the University of North Carolina to distribute the device.

## Authors' contribution

KL conceptualized the design and conduct of the experiment, performed data collection, data analysis and writing of the manuscript. MJ, MA, CZ assisted with data collection and writing the manuscript. MT contributed to the design and interpretation of the study and in the preparation of the manuscript. AJN conceptualized the design of the study, assisted with data analysis, interpretation and writing the manuscript. All authors read and approved the final manuscript.
